# Platysma transverse myocutaneous flap: a 21 case series of an overlooked reconstructive method for facial skin defects

**DOI:** 10.1016/j.bjorl.2019.10.011

**Published:** 2019-12-10

**Authors:** Fábio Muradás Girardi, Luiz Alberto Hauth, Aliende Lengler Abentroth

**Affiliations:** Hospital Ana Nery, Departamento de Otorrinolaringologia e Cirurgia de Cabeça e Pescoço, Santa Cruz do Sul, RS, Brazil

**Keywords:** Surgical flaps, Superficial musculoaponeutic system, Head and neck neoplasms, Skin neoplasms, Myocutaneous flap

## Abstract

**Introduction:**

Since the first report of a platysma transverse myocutaneous flap in 1977, few articles about this flap design have been added to the literature.

**Objective:**

Our aim is to describe our department’s experience with platysma transverse myocutaneous flap.

**Methods:**

A retrospective review of all patients undergoing platysma transverse myocutaneous flap reconstruction between 2011 and 2019.

**Results:**

There were 16 men and 5 women in this series. The mean patients’ age was 72.7 years old. In eight cases, we had wound complications, including four wound infections, one hematoma and three distal flap ischemia problems. Distal flap ischemia occurred only in cases that advanced beyond the midline and with length-to-width ratio equal to or over three to one. Neck dissection was performed in two of these three cases with ischemic complications.

**Conclusion:**

Several factors may influence platysma transverse myocutaneous flap survival. Usually a long and narrow flap, especially crossing the neck midline and associated with neck dissection are more prone to poor outcomes.

## Introduction

McGrath and Ariyan reported the first use of a platysma transverse myocutaneous flap (PTMF) in 1977 when covering a full-thickness burn of an ear.^1^ Even following years of expertise in free flaps, Ariyan returned to use the PTMF when needed to resurface moderately sized defects of cheek and preauricular areas. The platysma flap offered a better color IN addition to fulfilling the main requirements of a local flap: obtained locally, suitable thickness and permits direct closure of the donor site. In 1997, Ariyan reported another six consecutive cases, with only one partial skin paddle loss,^2^ followed by an update of two other successful cases in 2003.^3^ Since then, we found only four other articles describing their scarce experience on posteriorly based platysma myocutaneous flap, the major one from China in 2006, with 12 cases.^4^

In 1993, Martin et al. reported an alternative option to platysma flaps, based on branches of the submental artery, which allowed an increased rotation arc compared with the PTMF, with good esthetic and functional results.^5^ Although its execution was faster and simpler than microvascularized free flaps, it also usually requires careful dissection of the pedicle and additional care in the preservation of the mandibular branch of the facial nerve, which invariably increases the surgical time and the learning curve for its execution.^5^ In the following years, the development of the cervicosubmental keystone island flap incorporated the PTMF and the submental island flap reconstructive principles, without regard to identification of a specific perforator, and used the natural fasciocutaneous redundancy within the neck to raise the flap to the face.^6^ In a certain way, the large experience with both cervicosubmental and submental flaps substituted the PTMF in many head and neck surgery departments.

Despite the emergence of those new reconstruction techniques, the PTMF remained as an straightforward and short-learning-curve procedure, allowing the performance of large reconstructions, even with associated neck dissections, with the security of not necessarily cutting the skin at the base of the flap.^2^ It remained a useful flap and an important alternative in the reconstructive armamentarium, although the published experience is still scarce.

Here, we describe our experience with the PTMF to reconstruct facial skin and soft tissue defects. To our knowledge, it is the major case series reporting this flap design.

## Methods

The local institutional review board and a regional Research Ethics Committee approved the study protocol (CAAE: 93792318.4.0000.5304). A retrospective review of all patients undergoing PTMF reconstruction at a single institution was performed. Between 2011 and 2019, 21 transverse platysma designed flaps in 20 different patients were selected for analysis. Each flap was considered as an individual case for analysis purpose. Only patients undergoing head and neck oncologic surgery were considered. The collected data included patient demographics, comorbidities, pathology, flap dimensions and outcomes. For review purposes, the following terms were searched in PubMed: (transverse AND platysma AND flap) OR (posteriorly based platysma flap). Experience with PTMF was mentioned in six articles, totaling 38 published cases. Our group published another five cases,^7^, ^8^ not found among PubMed reviewed articles.

## Results

**Figure 1:**
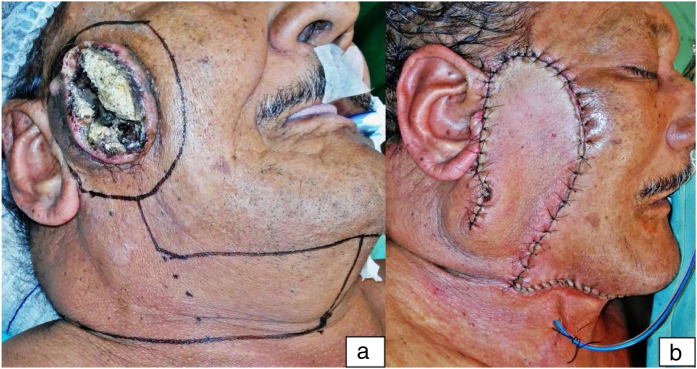
Illustrative case. Case “4″ from Table 1. In the first picture (a), a primary neglected cutaneous squamous cell carcinoma, deeply ulcerated by recent myiasis, and the surgical planning. In the second one (b), the immediate postoperative result.

Among the 21 cases, there were 16 men and 5 women. The mean patients’ age was 72.7 years old (range 40.4–95.5 years old). General information is summarized in Table 1. Cases 1, 2, 3, 5 and 11 were already published.^7^, ^8^ The most frequent tumor location was the parotid zone (14 cases). Histological analysis of specimens revealed a diagnosis of basal cell carcinoma in two patients, squamous cell carcinoma (SCC) in 12 patients, melanoma in four cases and the remaining three cases were a malignant subcutaneous pecoma and two basosquamous carcinomas. Only one case, an advanced SCC, had clinically suspicious neck lymph nodes at the first consultation in our department, but three other cases were submitted to elective superior neck lymphadenectomies for staging. In two cases, there was under-skin tunneling to reach the malar zone and the nasolabial region. In 16 cases, the flap reached the zygomatic arch; in one, the nasal ala zone and the remaining four, the inferior or medium portion of the ear. The mean length and width of the flap were 11.57 cm (range 6–18 cm) and 4.83 cm (range 3.5–10 cm), respectively. The mean length-to-width ratio was 2.42 (range 1.7–3.8). In 11 cases, the flaps advanced one to six centimeters beyond the midline. In eight cases, we had wound complications: four wound infections, one wound hematoma and three distal flap ischemias (two full thicknesses and one skin loss only). Postoperative wound infection compromised the flap integrity in case 10, associated with an intense inflammatory process in the preoperative period. Both case 10 and case 4 initially presented with wound myiasis, completely resolved at surgical act. Distal flap ischemia occurred only in cases that advanced beyond the midline and with length-to-width ratio equal to or over three. Two of them were submitted to associated neck dissection and one of them had associated under-skin tunneling. All our cases had many comorbidities. We calculated the Charlson Comorbidity Index (CCI) and the Adult Comorbidity Evaluation-27 (ACE-27) for all our patients. Results ranged from 2 to 11 when applying CCI, with 16 cases with CCI equal to or over 5. When applying ACE-27, eight cases were classified in Grade 2 and 10 in Grade 3. Comorbidities were distributed evenly over all cases, with or without complications. We did not have problems with closure of donor areas, even in younger individuals. Only in case 18 did we need a skin graft for closure of part of the neck defect. Esthetic and functional outcomes were satisfactory in all cases, even the complicated ones (Fig. 1).

**Table 1:** General information.

Name	Age	Sex	AP	Site	Tunneling	Distal flap reach	Length	Width	L/W	ABM	ND	Complications	CCI	ACE-27
Case 1	78.0	M	SCC	Malar	No	Zygomatic arch	13	6.5	2.0	2	No	No	6 pts	Grade 3
Case 2	41.0	M	BCC	Nasofacial	Yes	Nasal ala	14	4.5	3.1	1.5	No	No	2 pts	Grade 1
Case 3	78.0	M	SCC	Malar	No	Zygomatic arch	13	5	2.6	2	No	No	6 pts	Grade 3
Case 4	69.7	M	SCC	Parotid	No	Zygomatic arch	15	5	3.0	1.5	Level I‒II	Distal flap ischemia^a^	4 pts	Grade 3
Case 5	70.8	M	Pecoma	Parotid	No	Zygomatic arch	14	5	2.8	2	No	No	5 pts	Grade 2
Case 6	91.4	M	SCC	Bucal	No	Zygomatic arch	12	4	3.0	1	level I-II	Distal flap ischemia^a^	11 pts	Grade 3
Case 7	84.6	F	Melanoma	Bucal	No	Zygomatic arch	8	3.5	2.3	No	No	No	7 pts	Grade 2
Case 8	60.9	M	BCC	Parotid	No	Zygomatic arch	15	4	3.8	1	No	Hematoma	5 pts	Grade 2
Case 9	53.1	M	BSC	Parotid	No	Zygomatic arch	7	3.5	2.0	No	No	No	3 pts	Grade 1
Case 10	40.4	F	SCC	Malar	Yes	Zygomatic arch	13	4	3.3	4	No	Distal flap ischemia^a^	3 pts	Grade 3
Case 11	58.4	M	Melanoma	Parotid	No	Zygomatic arch	13	5	2.6	1.5	No	Wound infection	6 pts	Grade 3
Case 12	88.5	M	SCC	Parotid	No	1/2 of the ear	7	3.5	2.0	No	No	No	7 pts	Grade 2
Case 13	81.3	M	SCC	Parotid	No	1/3 of the ear	6	3.5	1.7	No	No	No	6 pts	Grade 2
Case 14	82.4	M	SCC	Parotid	No	1/2 of the ear	10	4	2.5	No	level I-II	Wound infection	6 pts	Grade 3
Case 15	77.7	F	Melanoma	Parotid	No	Zygomatic arch	8	4	2.0	No	No	No	2 pts	Grade 1
Case 16	95.5	F	SCC	Parotid	No	Zygomatic arch	15	7	2.1	2	No	No	8 pts	Grade 3
Case 17	80.5	M	SCC	Parotid	No	Zygomatic arch	8	4	2	No	No	No	7 pts	Grade 2
Case 18	69.9	M	SCC	Parotid	No	Zygomatic arch	18	10	1.8	6	Level I-II	Wound infection	11 pts	Grade 3
Case 19	66.8	M	SCC	Bucal	No	Zygomatic arch	13	6	2.1	No	No	No	5 pts	Grade 2
Case 20	76.5	F	Melanoma	Parotid	No	1/2 of the ear	10	4.5	2.2	No	No	No	7 pts	Grade 3
Case 21	80.4	M	BSC	Parotid	No	Zygomatic arch	11	5	2.2	No	No	Wound infection	7 pts	Grade 2

Age is expressed in years; Sex: M, Male; F, Female; AP, Anatomopathological result; BCC, Basal Cell Carcinoma; SCC, Squamous Cell Carcinoma; BSC: Basosquamous Carcinoma; Length and Width are expressed in centimeters; L/W, Length/Width; ABM, Advance Beyond Midline; ND, Neck Dissection.

a Only skin loss; Charlson Comorbidity Index (CCI); Adult Comorbidity Evaluation-27 (ACE-27).

## Discussion

Even with the widespread use of free flaps, regional flaps are still part of the head and neck surgeon’s armamentarium.^2^ We reported our department’s experience with PMTF. Scarce literature about this flap design is available. Besides Ariyan’s two classical articles, Peng and Su, from China, and Kocer, from Turkey, described another 12, 7 and 3 cases, respectively.^2^, ^3^, ^4^, ^9^, ^10^ In an article from Cleveland, USA, another seven cases were reported.^11^ Su et al. reported 12 relatively small-sized flaps (maximum length of 9 cm), with two partial necroses. No other detailed and specific information was available^4^ for comparison. Peng et al. described seven cases, only two for facial skin of soft tissue defects. They reported one total necrosis. The authors associated this complication with reconstruction of an oral defect, tunneling and a long portion of the distal flap beyond the midline (in this case, 3 cm).^9^ It is not clear if some cases reported by Su et al. are the same as Peng’s study because some authors share the same department. Kocer et al. reported three cases, with no distinction between vertical and transverse designed flaps when describing the results.^10^ Baur and Helman reported partial necrosis in four from seven cases, three of them with skin losses only, with muscle preservation, which did not represent a big complication when reconstructing oral defects. Distal full-thickness necrosis occurred in one case, this one associated with neck dissection.^11^

Ariyan’s reports are still the reference point for any study about PTMF because detailed information is available for comparison. Joining his nine published cases, the mean length, width and length-to-width ratio of the flaps were slightly larger than ours (14.88 cm [range 11–18 cm]; 5.66 cm [range 2.14–4.5 cm]; and 2.84 [range 2.14–4.5], respectively). As in our results, Ariyan observed a loss of the skin paddle in one of the two cases with length-to-width ratio greater than three.^2^, ^3^ These results suggest that PTMF survival might be determined by a constant length-to-width ratio. A similar explanation was classically attributed to the random cutaneous flaps,^12^ although nowadays new information suggests that other variables, such as thickness, may be implicated in flap survival.^13^ Another classical definition is that the skin paddle can be outlined across the midline of the neck, as long as more than half of the skin paddle is over the platysma muscle.^2^ Nevertheless, both our group and other authors^9^ observed distal ischemia complications among long flaps only, which advanced beyond the midline. Except for one case, the other ischemic complications of our series were associated with previous wound myiasis and a consequent intense inflammatory process in the surgical bed.

## Conclusion

We describe our experience with PTMF. It is an easy-to-perform flap, besides being associated with a low rate of severe ischemic complications. Apparently, several factors may influence this flap survival. Usually, a long and narrow flap, especially crossing the neck midline and associated with neck dissection, is more prone to poor outcomes. Wound infection complications or intense inflammatory process in the surgical bed may contribute to flap ischemia. Complications were distributed equally according to comorbidities scale results.

## Conflicts of interest

The authors declare no conflicts of interest.
